# Combination of Venetoclax and Midostaurin Efficiently Suppressed Relapsed t(8;21)Acute Myeloid Leukemia With Mutant KIT After Failure of Venetoclax Plus Azacitidine Treatment

**DOI:** 10.3389/fonc.2022.841276

**Published:** 2022-02-08

**Authors:** Zheng Li, Jun Wang, Shuai-Shuai Ge, Qiao-Cheng Qiu, Jia-Hui Du, Shuang-Shuang Shan, Xiang-Dong Shen, Chao-Ling Wan, Bin-Ru Wang, De-Pei Wu, Hui-Ying Qiu, Sheng-Li Xue

**Affiliations:** ^1^ National Clinical Research Center for Hematologic Diseases, Jiangsu Institute of Hematology, The First Affiliated Hospital of Soochow University, Suzhou, China; ^2^ Institute of Blood and Marrow Transplantation, Collaborative Innovation Center of Hematology, Soochow University, Suzhou, China; ^3^ Suzhou Key Laboratory of Medical Biotechnology, Suzhou Vocational Health College, Suzhou, China

**Keywords:** venetoclax, azacitidine, midostaurin, t(8;21), relapsed acute myeloid leukemia, KIT mutation, targeted therapy

## Abstract

Acute myeloid leukemia (AML) with t(8;21) is categorized as favorable-risk AML, but KIT mutations show a significantly poor prognostic impact in such patients. Persistent vulnerability to relapse is a major challenge in the treatment of this subtype of patients. Venetoclax is a BCL-2 selective inhibitor. The venetoclax+HMA strategy is also a notable salvage regimen that achieves good clinical outcomes in the treatment of relapsed or refractory (R/R) AML. However, in our clinical practice, we found that disease progressed rapidly even after venetoclax+azacitidine (AZA) therapy in two relapsed t(8;21) AML patients with KIT mutations. We report for the first time the therapeutic potential of venetoclax+midostaurin as a new combination therapy for relapsed t(8;21) AMLs with KIT mutations showing resistance to venetoclax+AZA therapy. Our ex vivo study also showed that midostaurin alone could inhibit proliferation and induce apoptosis of Kasumi-1 cells (e.g. Midostaurin induced G2 phase cell arrest, down-regulated p-KIT and BCL-2, while Bax protein levels were up-regulated) and observed a synergistic anti effect when the two drugs were combined. Our study shows that the venetoclax+midostaurin regimen may be a promising treatment option for R/R t(8;21) AML with KIT mutations.

## Introduction

Acute myeloid leukemia (AML) with t(8;21)(q22;q22.1)/AML1-ETO belongs to the core-binding factor (CBF) category of leukemia, which constitutes a distinct entity in the World Health Organization 2016 classification and comprises AML with inv(16)(p13.1q22) or t(16;16)(p13.1;q22)/CBFB-MYH11 (1). Because of the high CR rate of nearly 90% and high long-term survival rate, with a 5-year OS of almost 50% (2), AML with t(8;21) along with inv(16) is categorized as favorable-risk AML within the 2017 European LeukemiaNet (ELN) genetic risk stratification (3). However, the 5-year cumulative incidence of relapse (CIR) was more than 50% in this group of patients after high-dose cytarabine consolidation following CR (2). Furthermore, among the additional genetic events in the t(8;21) patients, KIT mutations appear to be the most common, with an occurrence rate of up to 31.8%, and mutations in exon 17 were the most frequent (4). KIT mutations, especially those in exon 17, show a significant poor prognostic impact in such patients (4). Allogeneic hematopoietic stem cell transplantation (allo-HSCT) is a theoretically curative approach for patients with KIT mutations. Unfortunately, even after HSCT, the 2-year CIR could approach 32%, and the 2-year leukemia-free survival (LFS) was only 55%; therefore, the long-term outcome still needs to be further improved (5). Persistent vulnerability to relapse is a major challenge in the management of this subtype of patients.

Venetoclax (VEN) is an oral BCL-2 selective inhibitor that has activity in a variety of hematologic malignancies (6). Clinical trials have demonstrated the benefit of venetoclax-based therapies. In November 2018, venetoclax was approved by the US Food and Drug Administration (FDA) in combination with hypomethylating agents (HMAs) or low-dose cytarabine for newly diagnosed AML or AML that is ineligible for intensive chemotherapy in adults aged 75 years and older (7). Venetoclax+HMAs is also impressive as a salvage regimen for relapsed or refractory (R/R) AML, achieving good clinical outcomes (8, 9). However, in our clinical practice, we found that the disease still progressed rapidly even after venetoclax+azacitidine (AZA) therapy in two relapsed t(8;21) AML patients with KIT exon 17 mutations.

Midostaurin (PKC412) is an inhibitor of multiple receptor tyrosine kinases (TKs). *In vitro*, midostaurin or its major active metabolites inhibit the activity of KIT (wild type and the D816V mutant), FLT3 (wild type, and ITD and TKD mutants), PDGFRα/β, VEGFR2 and members of the serine/threonine kinase protein kinase C family (10). Midostaurin was approved by the US FDA and the European Medicines Agency for both newly diagnosed FLT3-mutated AML and advanced systemic mastocytosis (SM) driven by KIT mutation (11). We explored the therapeutic strategy of venetoclax combined with midostaurin in two relapsed t(8;21) AML patients with KIT mutations who failed venetoclax plus azacitidine.

## Case Presentation

**Figure 1:**
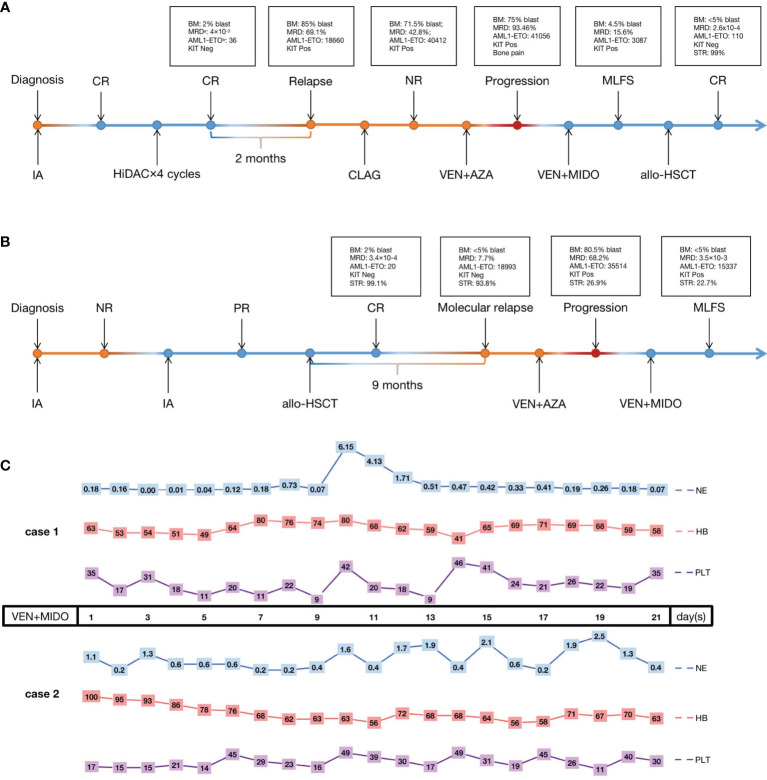
The treatment process of case 1 **(A)** and case 2 **(B)** and the hematological toxicity of VEN+MIDO therapy **(C)**. CR, complete remission; PR, partial remission; NR, non-remission; Neg, negative; Pos, positive; IA, cytarabine 100 mg/m^2^ continuous infusion d1-7, idarubicin 12 mg/m^2^ d1-3; HiDAC, cytarabine 2 g/m^2^ over 3 h every 12 h on d1-3; CLAG, cladribine 5 mg/m^2^ d1-5, cytarabine 2 mg/m^2^ d1-5, granulocyte-colony stimulating factor 5 μg/kg d0-5; VEN+AZA, venetoclax once daily (100 mg d1, 200 mg d2, 400 mg d3-28) and azacitidine 75 mg/m^2^ d1-7; VEN+MIDO, venetoclax once daily (100 mg d1-21) concurrent with voriconazole, midostaurin twice daily (50 mg d1-21); allo-HSCT, allogeneic haematopoietic stem cell transplantation; MLFS, morphologic leukemia-free state; NE, neutrophils count (×10^9^/L); HB, hemoglobin (g/L); PLT, platelet count (×10^9^/L). ^a^MRD, minimal residual disease detected by multiparameter flow cytometry. ^b^AML1-ETO, the presence of the AML1-ETO fusion gene calculated with standard materials, normalized with respect to the number of ABL1 transcripts and expressed as copy numbers per 1×10^4^ copies of abl.

Case 1 is a 33-year-old female diagnosed with AML. Her karyotype was 45, X, -X, t(8;21)(q22;q22) (8)/46, XX, idem (2), AML1-ETO was 39700 copies/10000 abl copies (AML1-ETO transcripts were normalized to 10^4^ abl copies), and KIT exon 17 and CSF3R mutations were detected. She was initially treated with a standard “3+7” regimen with idarubicin and cytarabine (IA) as induction with complete remission (CR) followed by four cycles of high-dose cytarabine (HiDAC) consolidation. However, 2 months after the last consolidation, the disease relapsed, and she failed to achieve CR again after salvage chemotherapy with CLAG (cladribine 5 mg/m^2^ d1-5, cytarabine 2 mg/m^2^ d1-5, G-CSF 5 μg/kg d0-5). BM analysis revealed 71.5% blasts and high AML1-ETO (40412 copies). This patient then received venetoclax+AZA for one cycle. After the treatment, the BM revealed 75% blasts, and the patient presented with symptoms of fatigue, lassitude and bone pain, which suggested disease progression. Then, one cycle of venetoclax+midostaurin chemotherapy (venetoclax 100 mg qd d1-21, midostaurin 50 mg bid d1-21) was administered, and the BM showed 4.5% blasts upon finishing the therapy. Then, the patient underwent allo-HSCT (**Figure 1A**).

Case 2 was a 35-year-old man. BM morphology and immunophenotyping identified AML. The karyotype was 46,XY,t(8;21)(q22;q22) (7), and AML1-ETO and KIT exon 17 mutations were detected. He was treated with a standard “7+3” IA regimen as induction with no remission (NR). A second IA regimen was given, and partial remission (PR) was achieved. Then, he received allo-HSCT therapy. Unfortunately, 9 months post-HSCT, he experienced molecular relapse with AML1-ETO up to 18993 copies; KIT mutation status was positive, and STR was 93.8%. Next, a venetoclax+AZA regimen was given, but the disease progressed rapidly, with 80.5% blasts in BM, and AML1-ETO reached 35514 copies after therapy. As in case 1, one cycle of venetoclax+midostaurin regimen was given, and the patient achieved a morphologic leukemia-free state (MLFS) after 3 weeks of treatment with<5% blasts in the BM. AML1-ETO was 15337 copies (**Figure 1B**). Unfortunately, this patient abandoned further treatment finally including second bone marrow transplant for financial problems. The last follow-up was 3 months later after venetoclax+midostaurin treatment, the patient is still alive.

The two patients tolerated venetoclax+midostaurin treatment well under supportive care. Grade IV hematological adverse events (AEs) occurred in both patients. Both cases received red blood cell (RBC) (10 U vs. 6 U) and platelet (5 U vs. 6.5 U) transfusions to treat low hemoglobin and platelet levels. G-CSF could be administered when the neutrophil count was below 1.0×10^9^/L. No pneumonitis, hepatotoxicity or tumor lysis syndrome occurred. Investigator-assessed AEs were graded according to the National Cancer Institute’s Common Terminology Criteria for Adverse Events (NCI CTCAE version 5.0) (**Figure 1C**).

## Discussion

**Figure 2:**
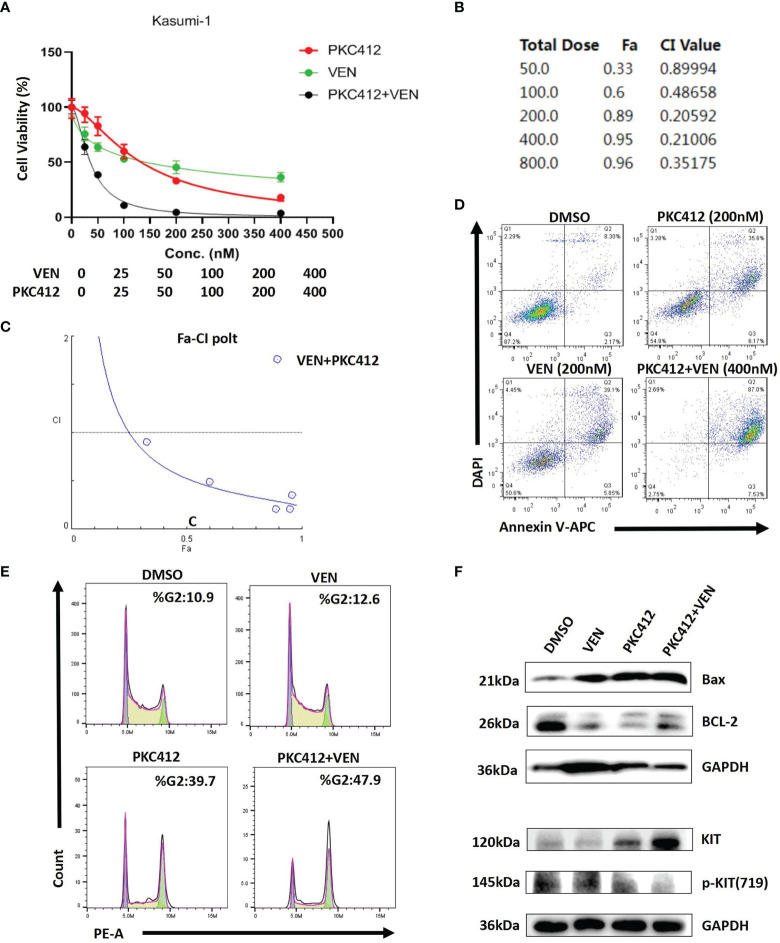
Venetoclax (VEN) synergizes with midostaurin (PKC412) to inhibit proliferation and induce apoptosis in Kasumi-1 cells. **(A)** The curve represents the dose-dependent effects of VEN and PKC412 on cell proliferation at 72 h. **(B, C)** Fa-CI plot and combination index (CI) values were calculated with CompuSyn software. CI < 1 indicates synergy, CI = 1 is additive, and CI > 1 indicates antagonism. The results showed that VEN combined with PKC412 had the most notable synergistic effect. **(D)** PKC412 induced apoptosis in Kasumi-1 cells and cooperatively induced apoptosis with VEN. Apoptosis was determined by Annexin-V/DAPI staining after Kasumi-1 cells were treated with PKC412 alone and in combination with VEN at the indicated concentrations for 72 h. **(E)** Kasumi-1 cells was treated with PKC412 and/or VEN at indicated concentration (500nM) for 72 h, and cell cycle analysis was performed by flow cytometry. **(F)** Kasumi-1 cells were exposed to PKC412 and/or VEN at 1μM for 72 h (BCL-2 and Bax) and 500nM for 8 h (KIT and p-KIT), then subjected to Western blotting. GAPDH was measured as a loading control.

Based on the excellent clinical trial results (9), venetoclax combined with HMAs is recommended as the first-line treatment of AML patients deemed ineligible for intensive therapy. In real-world clinical practice, this regimen is also associated with encouraging efficacy in R/R AML patients, with a CR+CRi rate of 61% (9). However, resistance to venetoclax-based combinations may rapidly ensue. Some studies have suggested that there are two categories of reasons underlying drug resistance: 1) Acquisition of high-risk gene mutations, such as FLT3 and RAS, that are involved in proliferation signals (12). 2) Involvement of two major anti-apoptotic BCL-2 family proteins, BCL-XL and MCL-1, which are determinants of resistance to venetoclax (13). However, neither of the patients we treated had the FLT3 and RAS mutations mentioned above. Considering that our patients harbored KIT mutations, which could also result in leukemia cell proliferation (14), we speculate that the activation of KIT mutation signals may be the cause of drug resistance. Most notably, in previous studies, midostaurin downregulated MCL-1 expression, which is a key determinant of both acquired and intrinsic resistance to venetoclax, and prevented venetoclax-induced upregulation of p-ERK, which is a key factor in proliferation signalling, conferring potential resistance to venetoclax. This implies the existence of a synergistic effect between venetoclax and midostaurin that could overcome the proposed two categories of resistance mechanisms. In fact, midostaurin and venetoclax synergistically induce apoptosis in AML cell lines and primary patient samples without KIT mutations (15). Our *ex vivo* study showed that venetoclax and midostaurin alone could inhibit proliferation and induce apoptosis of Kasuim-1 cells (harboring AML1-ETO fusion and KIT mutation) and observed a synergistic anti effect when the two drugs were combined (**Figures 2A–D**). We also found that midostaurin caused an arrest in G2 phase, the results of western blot showed that midostaurin significantly down-regulated BCL-2 and phospho-KIT(719), while Bax protein levels were up-regulated (**Figures 2E, F**).

## Conclusion

In conclusion, we provide the first report of the therapeutic potential of venetoclax+midostaurin as a new combination therapy for relapsed t(8;21) AML with KIT mutation showing resistance to venetoclax+AZA therapy. Taken together, our study shows that the venetoclax+midostaurin regimen may be a promising treatment option for R/R t(8;21) AML with KIT mutations.

## Data Availability Statement

The original contributions presented in the study are included in the article/supplementary material. Further inquiries can be directed to the corresponding authors.

## Ethics Statement

The studies involving human participants were reviewed and approved by the Ethics Committee of the First Affiliated Hospital of Soochow University. The patients/participants provided their written informed consent to participate in this study.

## Author Contributions

ZL, JW, and S-SG collected, analyzed data and wrote the manuscript. Q-CQ contributed to methodology and investigation. J-HD, S-SS, X-DS, and C-LW performed research and interpreted data. B-RW performed the experiments. D-PW, H-YQ, and S-LX conceived and designed the study. All authors read and approved the final manuscript.

## Funding

This work was supported by the grants from the National Natural Science Foundation of China (Grant No. 81970138), Translational Research Grant of NCRCH (Grant No. 2020ZKMB05), Jiangsu Province”333”project, Jiangsu Province Medical Youth Talent Program (Grant No. QNRC2016719), and Gusu Key Medical Talent Program (Grant No. GSWS2019007), Social Development-Clinical Frontier Project of Jiangsu Province (Grant No. BE2018652).

## Conflict of Interest

The authors declare that the research was conducted in the absence of any commercial or financial relationships that could be construed as a potential conflict of interest.

## Publisher’s Note

All claims expressed in this article are solely those of the authors and do not necessarily represent those of their affiliated organizations, or those of the publisher, the editors and the reviewers. Any product that may be evaluated in this article, or claim that may be made by its manufacturer, is not guaranteed or endorsed by the publisher.
